# Noradrenergic Locus Coeruleus‐CA3 Activation Alleviates Neuropathic Pain and Anxiety‐ and Depression‐Like Behaviors by Suppressing Microglial Neuroinflammation in SNI Mice

**DOI:** 10.1111/cns.70360

**Published:** 2025-03-25

**Authors:** Helin Zou, Weiyu Pu, Junli Zhou, Juan Li, Lulin Ma, Shuxian Wang, Chengxi Liu, Jing Mou, Xingfeng Liu, Tian Yu, Yiyong Wei, Haihui Xie, Song Cao

**Affiliations:** ^1^ Department of Anesthesiology, the Tenth Affiliated Hospital Southern Medical University (Dongguan People's Hospital) Dongguan Guangdong China; ^2^ Dongguan Key Laboratory of Anesthesia and Organ Protection Dongguan Guangdong China; ^3^ Key Laboratory of Anesthesia and Organ Protection of Ministry of Education (In Cultivation) Zunyi Medical University Zunyi Guizhou China; ^4^ Department of Anesthesiology Mianyang Hospital of Traditional Chinese Medicine Mianyang Sichuan China; ^5^ Department of Pain Medicine, the Tenth Affiliated Hospital Southern Medical University (Dongguan People's Hospital) Dongguan Guangdong China; ^6^ Affiliated Shenzhen Women and Children's Hospital (Longgang) of Shantou University Medical College (Longgang District Maternity & Child Healthcare Hospital of Shenzhen City) Shenzhen Guangdong China

**Keywords:** anxiety, depression, hippocampus, locus coeruleus, microglia, neuropathic pain, norepinephrine

## Abstract

**Objective:**

Neuropathic pain (NP) arises from neuroimmune interactions following nerve injury and is often accompanied by anxiety and depression. The aim of the study is to evaluate the effects of the noradrenergic locus coeruleus (LC), a key regulator of pain and emotional states, projects extensively to the hippocampus.

**Method:**

We investigated the effects of chronic NP on LC integrity and its projections to the hippocampal CA3 region in spared nerve injury (SNI) mice with behavioral tests, immunohistochemistry, neurochemical analyses, and Gq‐DREADD.

**Results:**

Chronic NP induced LC neuronal loss, reduced hippocampal norepinephrine (NE) release, and triggered microglial activation and neuroinflammation in CA3. Selective activation of LC‐CA3 noradrenergic neurons using Gq‐DREADD chemogenetics alleviated NP and comorbid anxiety‐ and depression‐like behaviors. This intervention suppressed microglial activation, decreased proinflammatory cytokines (TNF‐α and IL‐1β), and restored NE levels in CA3.

**Conclusion:**

Our findings highlighted the therapeutic potential of targeting LC‐CA3 projections to mitigate chronic NP and its neuropsychiatric comorbidities via modulation of hippocampal neuroinflammation.

## Graphical abstract

NP induces LC neuron loss; it also diminishes norepinephrine (NE) release and increases inflammatory activation of microglia and neuroinflammation in CA3 of the hippocampus. Selective activation of the LC‐CA3 noradrenergic neurons with Gq‐DREADD efficiently alleviates NP and anxiety‐ and depression‐like behaviors, and reduces microglial neuroinflammation in CA3 of mice 12 weeks post‐SNI.

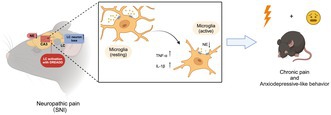

**Abbreviations**
AAV: Adeno‐associated virus
AD: Alzheimer's disease
ANOVA: one‐way analysis of variance
AR: adrenergic receptor
CCI: chronic constriction injury
CNO: clozapine N‐oxide
CNS: central nervous system
DREADD: designer receptors exclusively activated by designer drugs
ELISA: enzyme‐linked immunosorbent assay
EPM: elevated plus maze
Gi: inhibitory G proteins
IBA‐1: ionized calcium‐binding adapter molecule 1
LC: locus coeruleus
LTP: long‐term potentiation
MWT: mechanical withdrawal thresholds
NE: norepinephrine
NET: NE transporter
NP: neuropathic pain
OFT: open field test
PBS: phosphate buffer saline
PD: Parkinson's disease
PFA: paraformaldehyde
PFC: prefrontal cortex
qRT‐PCR: quantitative real‐time polymerase chain reaction
ROI: regions of interest
SNI: spared nerve injury
SNL: spinal nerve ligation
TH: tyrosine hydroxylase
TST: tail suspension test
TWL: thermal withdrawal latency

## 1 Introduction

Neuropathic pain (NP), characterized by nerve dysfunction due to neurological disorders, manifests as pain hypersensitivity or spontaneous pain [1]. Its pathogenesis involves complex neurotransmitter interactions and is frequently associated with comorbidities such as anxiety, depression, and cognitive deficits [2, 3, 4]. Despite its debilitating impact on quality of life, effective therapeutic strategies remain limited [5]. Consequently, the quality of life for NP patients is considerably compromised, underscoring the urgent need for effective therapeutic solutions.

Norepinephrine (NE), a key neurotransmitter regulating both nervous and immune systems [6, 7], is primarily synthesized in locus coeruleus (LC) neurons [8, 9]. LC degeneration during aging or neurodegenerative diseases (e.g., Alzheimer's and Parkinson's) contributes to cognitive decline and neuroinflammatory dysregulation [10]. This vulnerability leads to cognitive impairments and dysregulation of neuroinflammatory processes [11]. Notably, NP disrupts LC‐mediated NE signaling [12, 13], which is implicated in pain modulation and psychiatric comorbidities [14, 15], anxiety [16, 17, 18] and depression [19, 20]. Notably, NP affects LC function, including the release of NE from LC [15, 21, 22]. Chronic NP duration differentially impacts LC‐spinal cord and LC‐anterior cingulate cortex pathways [19, 23, 24], yet the long‐term effects on LC neuronal survival remain unclear.

Microglia, the CNS resident immune cells, drive neuroinflammation and central sensitization in chronic NP [25, 26]. Persistent microglial activation under sustained pain stimuli exacerbates anxiety and depression through adrenergic receptor‐rich regions like the hippocampus and prefrontal cortex [27, 28, 29, 30]. Noradrenergic signaling plays a critical role in modulating microglial activation states, regulating neural plasticity, and mediating behavioral adaptations [16, 27, 31, 32, 33].

The hippocampal CA3 region emerges as a key hub for NE‐mediated pain regulation. LC projections preferentially target CA3 compared to other hippocampal subregions [34], where NP induces TNF‐α‐mediated neuroinflammation [35] and dendritic atrophy in pyramidal neurons [36]. CA3 synaptic plasticity alterations correlate with NP‐associated memory dysfunction and affective disorders [37, 38, 39, 40, 41, 42, 43, 44]. Notably, CA3‐specific TNF‐α reduction alleviates both pain and depressive behaviors in NP models [43], suggesting NE‐modulated CA3 mechanisms may integrate pain and emotional processing.

This study investigates how chronic NP alters LC noradrenergic function and whether LC‐CA3 projections regulate NP‐related neuroinflammation and affective behaviors. We hypothesize that selective activation of LC‐CA3 noradrenergic pathways will mitigate NP, suppress CA3 microglial activation, and improve anxiety/depression‐like behaviors in mice.

## 2 Materials and Methods

### 2.1 Animals

This study was conducted in accordance with the ethical guidelines and received formal approval from the Laboratory Animal Welfare and Ethics Committee of Zunyi Medical University (ZMU21‐2210‐002) and in compliance with the guidelines provided in the Guide for the Care and Use of Laboratory Animals [45]. In this study, due to the long duration of pain modeling, and considering the potential impact of hormonal changes in female mice, female mice were not used. However, the gender of mice influences both pain perception and treatment [46]; the results and conclusions of this study are only applicable to male mice. Male C57/BL6 mice (8–12 weeks old) and weighing 25–30 g were procured from Biotechnology (Changsha, China). Transgenic tyrosine hydroxylase (TH)‐cre mice were generously provided by professor Wei Shen of Shanghai University of Science and Technology. Housing conditions for the mice consisted of three to four animals per cage, with a constant room temperature of 23°C ± 2°C, a relative humidity of 55% ± 2%, and a 12:12‐h light/dark cycle. Food and water were available ad libitum, and all mice were acclimated to the environment for a period of 1 week before the commencement of the experiments.

### 2.2 Methods

#### 2.2.1 Identification of TH‐Cre Transgenic Mice

The TH‐cre mouse line was generated through targeted insertion of the cre recombinase gene into tyrosine hydroxylase (TH)‐expressing neurons, enabling specific genetic manipulation of TH‐positive neuronal populations [47], particularly noradrenergic neurons in LC. At postnatal day 14, mice underwent ear punching for permanent identification prior to genotyping procedures. Genomic DNA was isolated using a lysis buffer system (Buffer L: Protease Plus = 50:1 v/v ratio) through a 2‐h incubation at 55°C, followed by centrifugation at 12,000 *g* for 10 min. After centrifugation to obtain the supernatant, 1 μL of this solution was added to 19 μL of the amplification system (composition of amplification system: ddH2O: 2× Taq Master Mix: TH‐cre‐F: TH‐cre‐MT‐R: TH‐cre‐WT‐*R* = 7.5:10:0.5:0.5:0.5:0.5), mixed thoroughly, and then subjected to a 2‐h polymerase chain reaction (PCR) amplification. Six microliters of the amplified product were electrophoresed on an agarose gel at 120 V for 60 min, followed by gel exposure for genotyping to identify mice with the TH‐cre genotype for subsequent experiments. Primer sequences: Common reverse: 5′‐TGGGATATGTTGTACAAAAAGAACT‐3′; Wild type reverse: 5′‐TTAGCTAGCTGAACTCTAAATGAGC‐3′; Mutant reverse: 5′‐CACGGGCAACTTTCC‐3′.

#### 2.2.2 Spared Nerve Injury (SNI) Model

Mice were anesthetized with 2%–3% (v/v) isoflurane and maintained on a thermostatic heating pad. Under aseptic conditions, a 1 cm longitudinal skin incision was made proximal to the left knee joint. The biceps femoris muscle was bluntly dissected to expose the sciatic nerve and its branches. The tibial and common peroneal nerves were transected, leaving the peroneal nerve intact. The severed nerve stumps were ligated with 6–0 chromic gut sutures, followed by layered wound closure. Sham‐operated mice underwent identical procedures except for nerve transection or ligation.

#### 2.2.3 Chemogenetics Model Establishment

Designer Receptor Exclusively Activated by Designer Drug (DREADD) [48] was utilized to selectively modulate LC‐CA3 noradrenergic projections. Forty‐eight adult male TH‐cre mice (20–25 g) were anesthetized with 2% isoflurane and immobilized in a stereotaxic frame after loss of pedal reflexes. Following scalp disinfection using iodophor, a cranial incision was made, the dura mater was carefully cleared, and cranial bone sutures were identified. The cranial surface of the mouse was leveled using the fontanel (Bregma) and the posterior fontanel (Lambda) as reference points, ensuring that the height difference between the anterior and posterior aspects did not exceed 0.02 mm. Based on the Paxinos mouse brain atlas [49], bilateral LC coordinates were determined (AP: −5.38 mm, ML: ±1.03 mm, DV: −3.9 mm). Subsequently, cranial bone was removed at the LC target site by drilling. A microinjection needle was used to inject the activating virus rAAV‐Ef1α‐DIO‐hM3D (Gq)‐mCherry‐WPREs at a rate of 150 nL/min. Following a 5‐min pause at the extraction site, the virus was bilaterally injected into the LC at a rate of 20 nL/min (80 nL on each side), with the microinjection needle remaining in place for an additional 10 min at the end of the injection. Subsequently, the mice were placed in a warm environment and allowed to regain consciousness and recover. Prior to conducting subsequent behavioral experiments, the mice were positioned in the hippocampal CA3 region with the following coordinates: anteroposterior [AP]: −2.10 mm, medial‐lateral [ML]: ±2.35 mm, and dorsal‐ventral [DV]: −2.35 mm. Microinjections were performed bilaterally in the CA3 region of the hippocampus, administering either 0.5 μL of 1 μg/μL Clozapine‐N‐Oxide (CNO) [50] or 0.5 μL of normal saline (NS) at a rate of 0.5 μL/min. This procedure was implemented to specifically modulate the LC‐CA3 projecting neurons.

#### 2.2.4 Single‐Unit Firing Recordings of LC Neurons

Mice were anesthetized with 2% isoflurane until abolition of withdrawal reflexes and secured in a stereotaxic frame. After surgical exposure of the skull, screws at the viral injection site were removed. A monopolar tungsten electrode (WE3003X; Microprobes for Life Sciences, USA) was advanced into the locus coeruleus (LC) via micromanipulator, targeting a depth of 3500 μm below the dura mater. Upon reaching 3000 μm, insertion speed was reduced to 1 μm/s. Extracellular action potentials were monitored in real time, with LC noradrenergic neuron identity confirmed through characteristic firing patterns. Baseline neuronal activity was recorded over 20 min.

Mechanical stimuli (1 g von Frey filament application and pinch) were applied to the ipsilateral hindpaw. Neuronal activity was recorded in vivo using a multi‐channel fiber photometry system (Tinkertech, China) interfaced with the electrode, with signals processed and stored via Spike2 software (CED, UK).

#### 2.2.5 Fiber Photometry Recordings

Fiber photometry was implemented to track NE dynamics in the hippocampal CA3 region ipsilateral to SNI. Recombinant AAV‐hsyn‐GRAB‐NE2m (3.1) (BrainVTA, PT‐2393, Wuhan, China) was made to record NE release in the hippocampal CA3. According to the above spatial coordinates, the bilateral hippocampal CA3 regions associated with pain processing were targeted, and 0.5 μL of rAAV‐hsyn‐GRAB‐NE2m (3.1) was carefully injected at a controlled rate. An optical fiber was implanted and securely fixed to the skull. Following a 3‐week viral expression period, the NE signal recordings were initiated.

Baseline fluorescence (F0) was established through 200‐s recordings in awake, unrestrained mice. Mechanical stimulation of the ipsilateral hindpaw with a 1 g von Frey filament was applied while monitoring real‐time fluorescence (F). NE‐dependent signaling was quantified as ΔF/F = (F‐F0)/F0 to assess LC‐mediated NE release.

#### 2.2.6 Assessment of Mechanical Allodynia and Thermal Hyperalgesia

For mechanical allodynia, von Frey filaments with ascending forces expressed in grams were used [51]. Mice were positioned on a mesh suspension platform and allowed to acclimate to the environment for 30 min. Mechanical withdrawal thresholds (MWT) were measured using a von Frey filament kit (Danmic Global, USA) with the up‐down method [52].

For thermal hyperalgesia, mice were placed in individual plexiglas chambers (20 × 20 × 15 cm) on a glass platform maintained at 25°C. They were given 30 min to adapt to the environment. Thermal withdrawal latency (TWL) was determined using a plantar radiant heat pain tester (IITC, Wood Dale, IL, USA). The device was activated by positioning the heat source directly beneath the left hind paw. The mice were observed for the exact time at which they withdrew their hind paws due to the heat. A 20‐s cutoff time was set to prevent tissue damage. The left paws were tested at 5‐min intervals for a total of five trials. The average of five tests, with a 5‐min interval between each trial, was considered the thermal withdrawal latency [15].

#### 2.2.7 Assessment of Anxiety‐ and Depression‐Like Behavior

All behavioral assessments were conducted after ≥ 24 h habituation to the testing environment under standardized conditions (20 lx illumination). During the testing sessions, the behavior of the animals was recorded using a video tracking system and subsequently analyzed offline. The room was maintained under dim lighting conditions, approximately 20 lx, to minimize any potential anxiety in the animals. Importantly, the experimenter remained blinded to the group identity throughout the experiment and quantitative analyses.

##### 2.2.7.1 Open Field Test (OFT)

Mice were placed in one corner of an open field apparatus consisting of a square area (25 × 25 cm^2^) bordered by a peripheral area (50 × 50 × 60 cm^3^). The mice were allowed to freely explore their surroundings, and their movement trajectories were recorded for 5 min using EthoVision XT software (Noldus). The software recorded the number of entries into and the time spent in the central area. Between tests, the apparatus was cleaned with 75% ethanol to remove any olfactory cues.

##### 2.2.7.2 Elevated Plus Maze (EPM) Test

The EPM consisted of a central platform (6 × 6 cm^2^), two enclosed arms (30 × 6 × 20 cm^3^), and two opposing open arms (30 × 6 cm^2^). The maze was positioned 100 cm above the floor, and the open arms were illuminated at approximately 20 lx. A mouse was placed on the central platform facing a closed arm and allowed to explore the maze for 5 min. The time spent in the open arms and the number of entries into the open arms were analyzed using EthoVision XT software. The maze was cleaned with 75% ethanol between tests to eliminate olfactory cues.

##### 2.2.7.3 Tail Suspension Test (TST)

In the TST, mice were gently suspended by a paper clip attached to the last 5 mm of their tails for a duration of 6 min. The mice were tested one cage at a time, and their behavior was recorded on video. Videos were subsequently analyzed by a trained rater who remained blinded to the experimental condition.

#### 2.2.8 Immunofluorescence

Mice were anesthetized with 2% isoflurane and perfused with phosphate‐buffered saline (PBS, 0.1 M, pH 7.4) followed by 4% paraformaldehyde (PFA). Subsequently, the ipsilateral spinal cord and sciatic nerve were promptly excised, and the tissues were transferred to 30% sucrose for dehydration. Once the tissues had sunk to the bottom of the sucrose solution, serial 30‐μm‐thick transverse sections were cut using a CM3050 S cryostat (Leica Microsystems, Wetzlar, Germany) at −20°C, collected in PBS containing 0.3% Triton X‐100 (three washes of 5 min each). The sections were then subjected to blocking with a solution of 1% BSA and 0.3% Triton X‐100 in PBS for a duration of 2 h. Subsequently, the sections were incubated with primary antibodies, including rabbit anti‐IBA‐1 (ionized calcium binding adapter molecule 1) (1:1000, Kaz, Osaka, Japan), mouse anti‐TNF‐α (1:1000, Abcam, Cambridge, UK), and mouse anti‐IL‐1β (1:1000, Santa Cruz, Dallas, USA) overnight at 4°C. Following three washes of the primary antibodies with PBS containing 0.3% Triton X‐100 (each for 5 min), the sections were treated with secondary antibodies: goat anti‐rabbit IgG H&L (1:1000; Abcam, Cambridge, UK) and goat anti‐mouse IgG H&L (1:1000; Abcam, Cambridge, UK). This incubation occurred for 2 h at room temperature in the absence of light. Subsequently, the sections were mounted onto glass slides and imaged using a fluorescence microscope (Olympus, Tokyo, Japan). Image J software (v.1.8, NIH, Washington, USA) was employed to calculate the proportion of cell fluorescence. For the quantification of IBA‐1, IL‐1β, and TNF‐α expression, Image J was used to outline hippocampal images with size‐standardized regions of interest (ROIs) [15, 53], and the percentage of fluorescent positive regions was determined with Image J. The total number of cells in each ROI was quantified using ImageJ, while double‐positive cells (yellow in merged images) were manually identified and counted by a lab technician who was blinded to the experimental design and group assignments [53].

#### 2.2.9 Enzyme‐Linked Immunosorbent Assay (ELISA)

Mice were transcardially perfused with ice‐cold PBS under deep anesthesia. Brains were rapidly extracted, snap‐frozen, and stored at −80°C. For the ELISA experiments, the brain tissue was homogenized in 0.1 M PBS with a protease inhibitor cocktail (20 μL/100 mg tissue, P8340, Sigma, Israel). The homogenate was then centrifuged at 1000 rpm, 4°C for 10 min, and the resulting supernatant was collected. Subsequently, the total protein content was determined using a BCA assay kit (23,225, Thermo Fisher, USA). The concentration of the supernatant was normalized to the protein content, and the levels of TNF‐α and IL‐1β (Beyotime, Shanghai, China), as well as NE (Elabscience, Wuhan, China), were measured using ELISA kits according to the manufacturer protocols, with absorbance measured at 450 nm.

#### 2.2.10 Quantitative Real‐Time Polymerase Chain Reaction (qRT‐PCR)

The hippocampus from mouse brains was isolated, and total RNA was extracted using RNAiso Plus (TaKaRa, Shiga, Japan). Genomic DNA was removed, and cDNA was synthesized from the total RNA using the Ten PrimeScript RT reagent kit with gDNA Eraser (TaKaRa, Shiga, Japan). Quantitative real‐time PCR (qRT‐PCR) was performed using the CFX96 Real‐Time PCR Detection System (Bio‐Rad, California, USA) with TB Green Premix Ex Taq II (TaKaRa, Shiga, Japan). Reaction conditions: 95°C/30 s initial denaturation; 40 cycles of 95°C/5 s denaturation and 60°C/30 s combined annealing/extension; concluding with melt curve analysis (65°C–95°C, 0.5°C increments) to confirm amplification specificity. Primer sequences are listed in Table 1. Data analysis was carried out using the $2^{-\Delta \Delta C_{t}}$ method.

**TABLE 1:** Primer sequences.

Genes (encoding protein)	Primer sequences
Cd68 (CD68)	
Forward	5′‐ACAGGCAGCACAGTGGACATTCA‐3′
Reverse	5′‐AGAAACATGGCCCGAAGTGTCCC‐3′
Il1b (IL‐1β)	
Forward	5′‐CTGCTTCCAAACCTTTGACCTG‐3′
Reverse	5′‐TGGGCTCTTCTTCAAAGATGAA‐3′
Tnf (TNF‐α)	
Forward	5′‐CTGAACTTCGGGGTGATCGG −3′
Reverse	5′‐GGCTTGTCACTCGAATTTTGAGA −3′
Il6 (IL‐6)	
Forward	5′‐CCACGATTTCCCAGAGAACAT −3′
Reverse	5′‐TCCATCCAGTTGCCTTCTTGG −3′
Mrc1 (CD206)	
Forward	5′‐GACCTTGGACTGAGCAAAGGGG‐3′
Reverse	5′‐AGAGCGTCCACGCAGCGCTTGT‐3′
Gapdh (GAPDH)	
Forward	5′‐GCTCATGACCACAGTCCATGC‐3′
Reverse	5′‐CAGATCCACGACGGACACATTG‐3′

#### 2.2.11 Statistical Analysis

Quantitative data were expressed as mean ± standard error of the mean (SEM) throughout. Statistical analysis was performed using GraphPad Prism 8.0 (GraphPad Software, La Jolla, USA) and SPSS 19.0 (IBM, Chicago, IL, USA). All data underwent normality testing using the Shapiro–Wilk test. Unless otherwise specified, all datasets in this study were normally distributed. One‐way analysis of variance (ANOVA) was employed for comparisons between multiple groups at the same time point. Two‐way repeated measures ANOVA was used for comparisons within the same group among different time points. Post hoc analysis was conducted using Tukey's test for multiple comparisons between two groups. Analysis of immunofluorescence images was performed using Image J (v.1.8).

## 3 Results

### 3.1 SNI Induced NP and Anxiety‐ and Depression‐Like Behaviors

**FIGURE 1:**
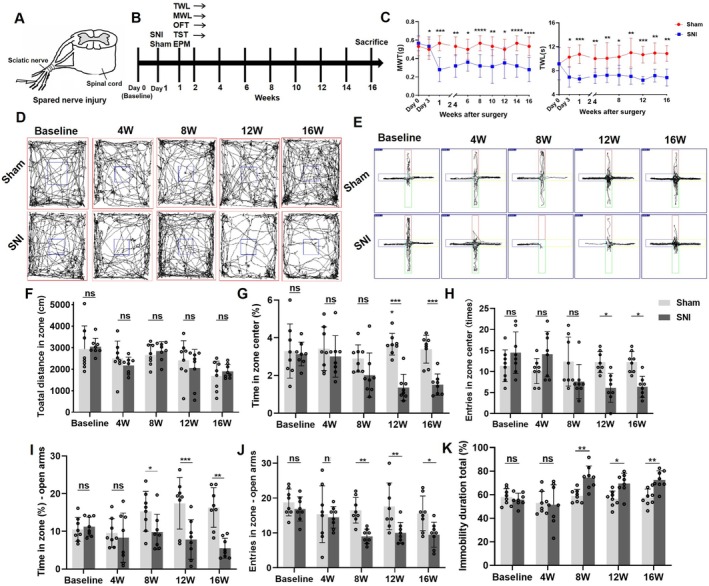
Behavioral changes and anxiety‐ and depression‐like behavior evaluation in SNI mice. (A) Schematic of SNI model. (B) Behavioral assessment timeline. (C) Changes of MWT and TWL in mice after SNI. (D) Behavior tracks of two groups of mice in OFT. (E) Behavior tracks of two groups of mice in the EPM test. (F) Total distance covered by mice in the OFT. (G, H) Percentage of time spent in the center of the OFT and number of entries into the center. (I, J) EPM time spent in the open arm and number of entries into the open arm. (K) Immobility time during the last 4 min of the TST. *p < 0.05, **p < 0.01, ***p < 0.001, ****p < 0.0001.

MWT and TWL were continuously tested at 3 days, 7 days, 1 week, 2 weeks, 4 weeks, 6 weeks, 8 weeks, 10 weeks, 12 weeks, 14 weeks, and 16 weeks after SNI modeling (Figure 1A,B). The results showed that MWT and TWL of the SNI group were significantly reduced compared with the Sham group from 3 days after modeling and maintained for up to 16 weeks (Figure 1C). Additionally, to investigate the occurrence of behavioral changes in the mice, the OFT, TST, and EPM tests were conducted before modeling and at 4 weeks, 8 weeks, 12 weeks, and 16 weeks after modeling (Figure 1D–J). For the total distance of OFT, no significant differences were observed from 4 to 16 weeks post‐SNI between the SNI and Sham groups (Figure 1F). However, from 12 weeks post‐SNI, the SNI group mice exhibited a reduction in the proportion of time spent in the center of the open field compared with the Sham group mice (Figure 1G), along with a decrease in the number of entries into the center of the open field (Figure 1H). In the EPM test, beginning at 8 weeks post‐operation, the SNI mice displayed a decrease in the proportion of time spent in the elevated open arm (Figure 1I) and a reduction in the number of entries into the open arm (Figure 1J). These findings indicated that the SNI mice developed anxiety‐like behaviors 8 weeks after SNI. Furthermore, starting at 8 weeks post‐SNI, the TST results revealed an increased proportion of immobility time in the last 4 min of the test for the SNI group mice compared with the Sham group mice (Figure 1K), indicating the presence of depression‐like behaviors in the SNI group.

### 3.2 Chronic NP Induced LC Neuron Loss, and Decreased NE Transporter (NET)‐Positive Nerve Fibers in the CA3, and Reduced NE Production in the Hippocampus

**FIGURE 2:**
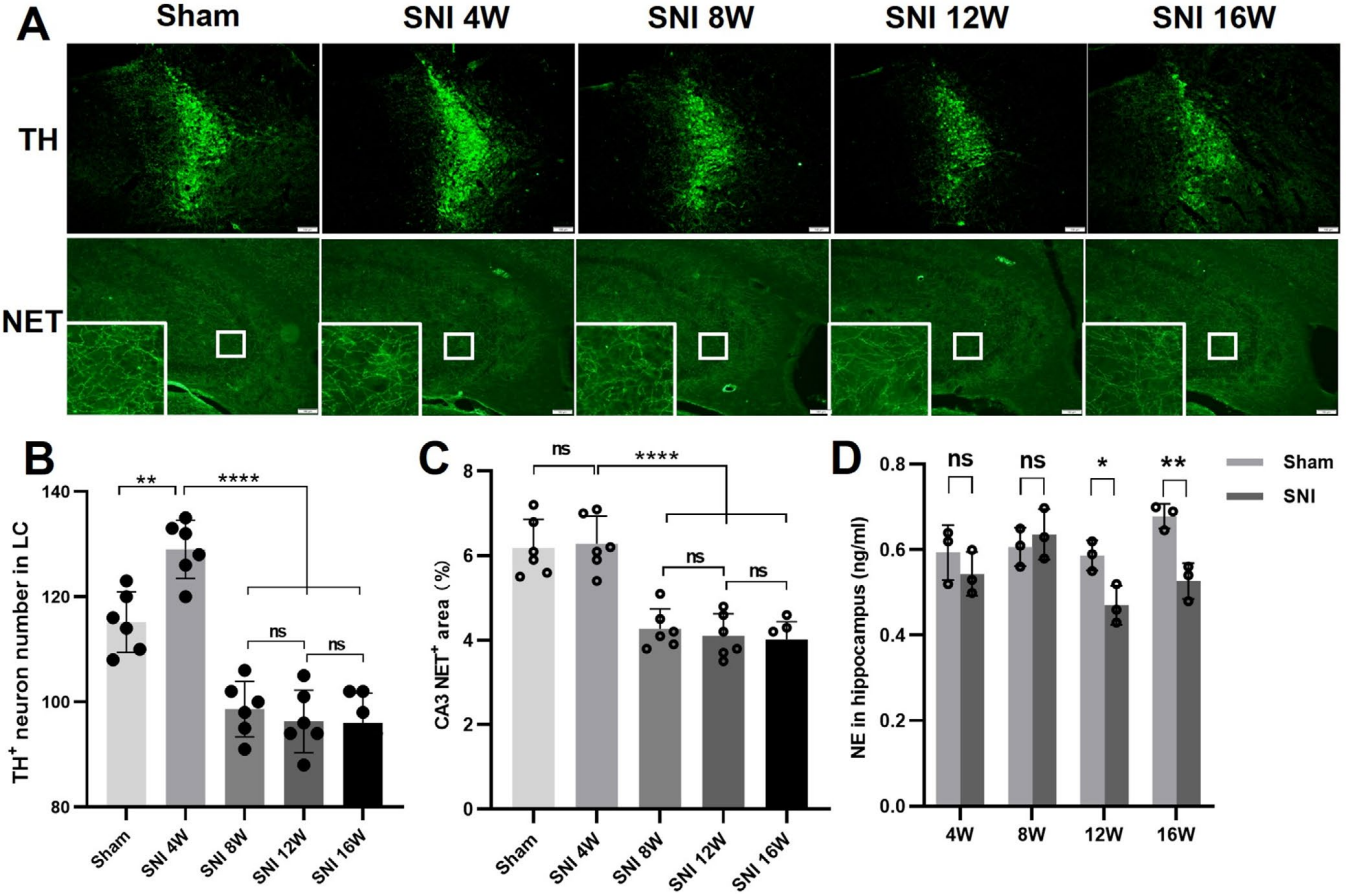
Changes of TH^+^ neuron number in LC, NET^+^ nerve fibers in the CA3 region of the hippocampus, and NE content in hippocampus. (A) Green fluorescence indicating LC TH^+^ neurons. (B) Green fluorescence indicating NET^+^ nerve fibers. (C) Long‐term chronic pain caused a decrease in the number of TH^+^ neurons in LC and sparser NET^+^ nerve fibers in the CA3 region of the hippocampus. (D) ELISA detection revealed that 12 and 16 weeks post‐SNI, NE content reduced significantly in SNI‐ipsilateral hippocampus of SNI mice compared with that of Sham‐operated mice. 4 V: Fourth ventricle. Bar = 100 μm. **p < 0.01, ****p < 0.0001, ns = not significant.

Quantitative immunofluorescence analysis was used to evaluate the number of TH^+^ neurons in the SNI‐ipsilateral LC. The results showed that the number of TH^+^ neurons in the LC increased 4 weeks after SNI compared with the Sham group, while at 8 W, 12 W, and 16 W post‐SNI, the number of TH^+^ neurons in the LC was significantly reduced compared with the Sham group and 4 W post‐SNI group (Figure 2A,B). At the same time, no significant change in the fluorescence density of NET^+^ nerve fibers in the SNI‐ipsilateral CA3 region was observed in the 4 W SNI group compared with the Sham group. However, at 8 W, 12 W, and 16 W post‐SNI, the fluorescence density of NET^+^ nerve fibers in the CA3 region was significantly decreased compared with the Sham group and 4 W SNI group (Figure 2A,C). ELISA detection revealed that 12 and 16 weeks post‐SNI, NE content was reduced in the SNI‐ipsilateral hippocampus of SNI mice compared with that of Sham ones (Figure 2D).

### 3.3 Chronic NP Induced Inflammatory Activation of Microglia in the Hippocampal CA3 Area

**FIGURE 3:**
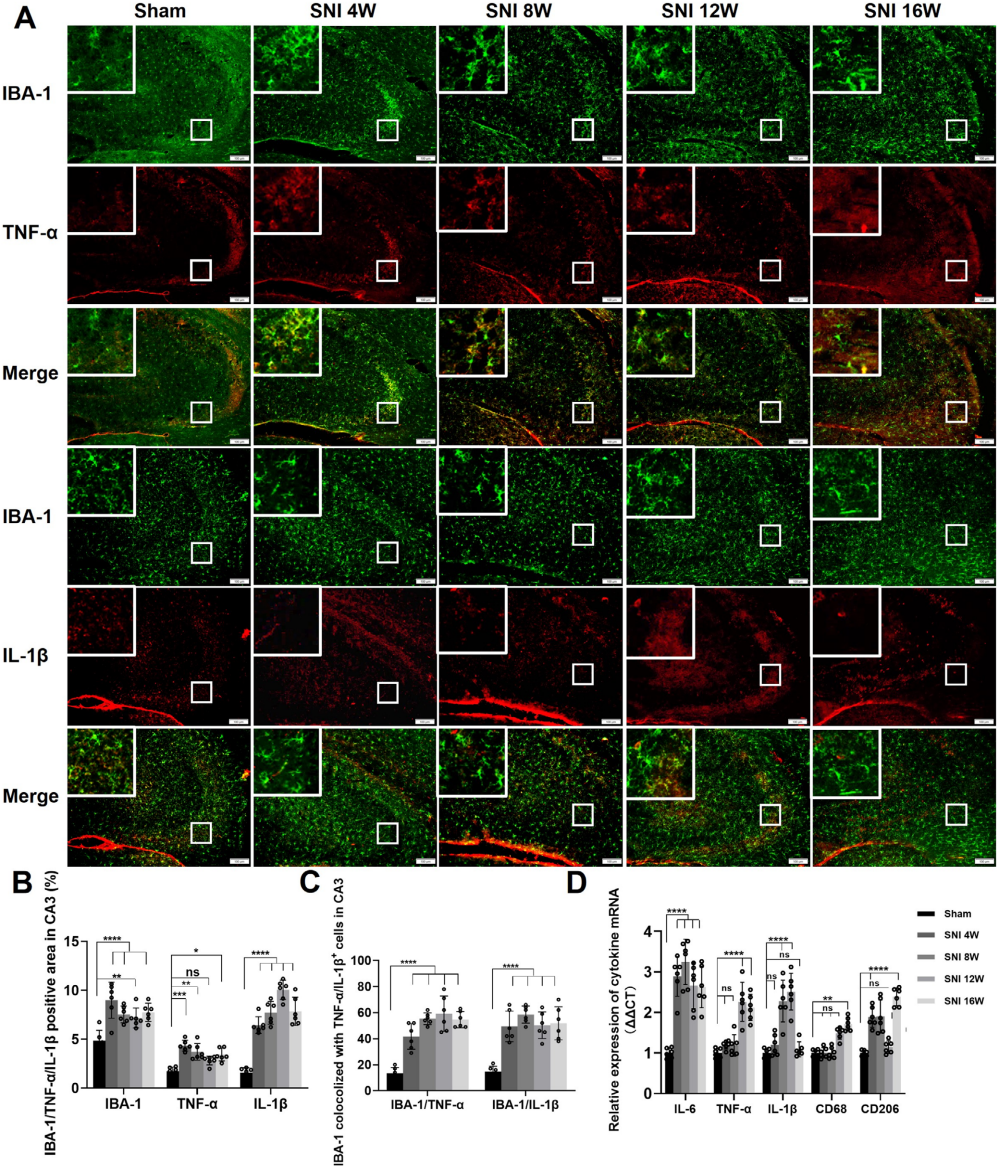
Microglial activation and neuroinflammation in hippocampal CA3 of SNI mice. (A) Sham and SNI mice of different pain durations were co‐stained with IBA‐1, TNF‐α, and IL‐1β. (B) SNI mice of different pain durations exhibited an increased fluorescence positive area for IBA‐1, TNF‐α, and IL‐1β in the SNI‐ipsilateral CA3 region compared with Sham mice. (C) SNI mice of different pain durations showed an increased co‐localization of IBA‐1 with TNF‐α or IL‐1β in the CA3 region compared with the Sham group. (D) Compared with the Sham group, hippocampal IL‐6 mRNA levels in the SNI group were significantly increased at 4 W, 8 W, 12 W, and 16 W after SNI surgery; TNF‐α mRNA levels were significantly increased at 12 W and 16 W after SNI; IL‐1β mRNA levels were significantly increased at 8 W and 12 W after SNI; CD68 mRNA levels were significantly increased at 16 W in SNI mice; IL‐1β mRNA levels were significantly increased at 2 W, 12 W, and 16 W in the SNI hippocampus; CD206 mRNA levels were significantly increased at 4 W, 8 W, and 16 W after SNI. Scale bar = 100 μm. *p < 0.05, **p < 0.01, ***p < 0.001, ****p < 0.0001.

Immunofluorescence analysis of the SNI‐ipsilateral hippocampal tissues revealed chronic NP‐induced microglial activation in the CA3 region, as evidenced by increased IBA‐1 immunoreactivity. This activation was accompanied by sustained upregulation of pro‐inflammatory mediators TNF‐α and IL‐1β (Figure 3A–C). Notably, the fluorescence of inflammatory cytokines was found to be colocalized with IBA‐1, suggesting that these inflammatory cytokines originated from microglia (Figure 3A–C). Subsequently, the mRNA expression levels of inflammatory cytokines and biomarkers (IL‐6, TNF‐α, IL‐1β, CD68, CD206) in the mice SNI‐ipsilateral hippocampal tissue were evaluated using RT‐qPCR. We found a continuous increase in IL‐6 levels after 4 weeks of SNI, an elevation of TNF‐α levels at 12 weeks after SNI, an upregulation of IL‐1β levels at 8 and 12 weeks after SNI, an increase in CD68 levels after 16 weeks of SNI, and upregulated CD206 levels at 4 and 16 weeks after SNI (Figure 3D).

### 3.4 LC Neurons Projected to CA3 in TH‐Cre Mice and Could Be Activated by Injecting CNO at CA3

**FIGURE 4:**
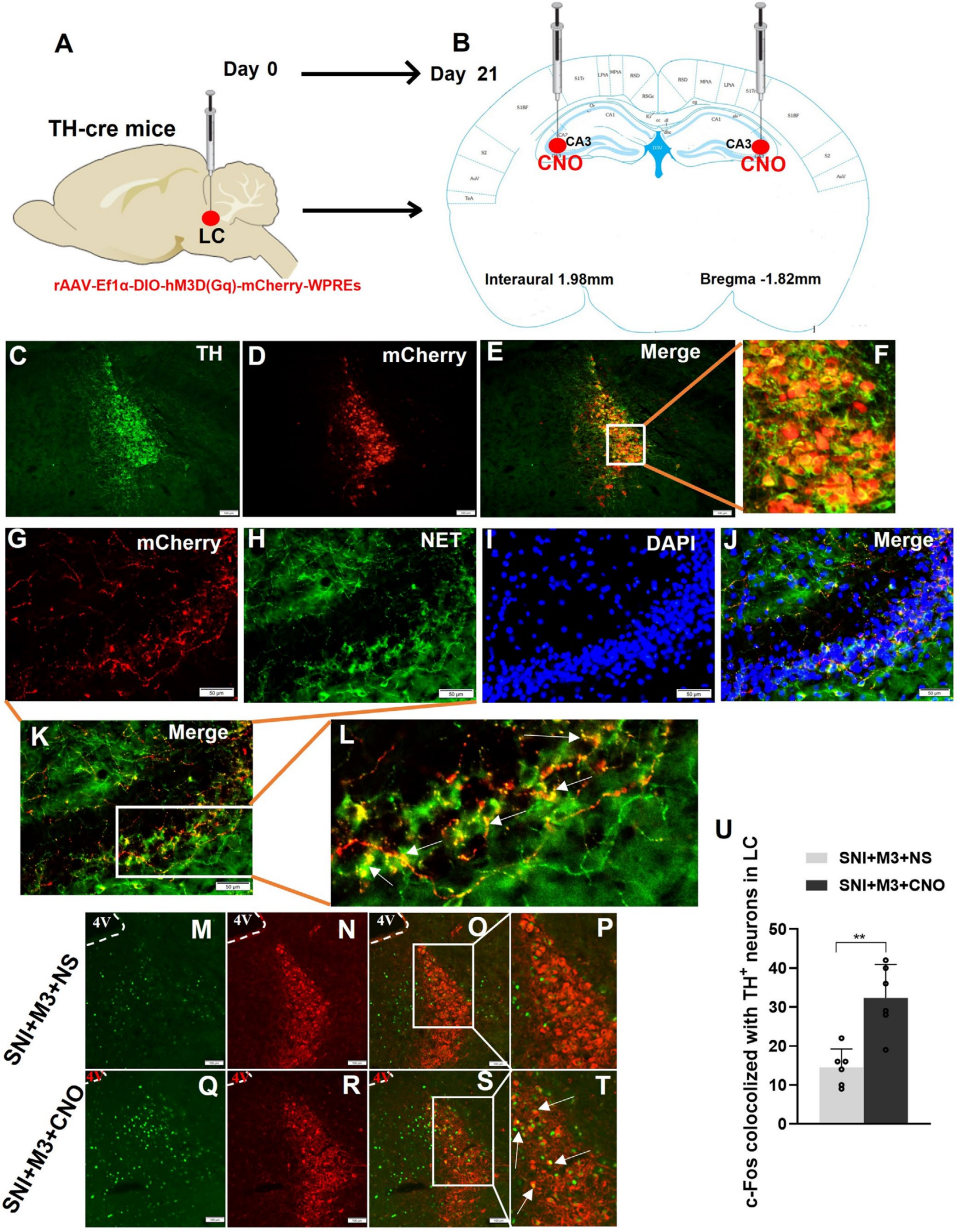
LC neurons projected to CA3 in TH‐cre mice and could be activated by injecting CNO at CA3. (A) Injection of Gq‐DREADD rAAV into the LC of TH‐cre mice. (B) CNO was injected into the bilateral hippocampus 21 days after adeno‐associated virus (AAV) injection. (C) Green fluorescence indicates LC TH^+^ neurons. (D) Red fluorescence (mCherry) showing successful transfection of rAAV‐Ef1α‐DIO‐hM3D(Gq)‐mCherry‐WPREs in LC. (E, F) Colocalization of TH^+^ neurons with mCherry. (G) Red fluorescence (mCherry) demonstrating TH^+^ neurons projects to CA3 region of the hippocampus. (H) Green immunofluorescence indicating hippocampal NET^+^ nerve fibers. (I) DAPI staining of the hippocampal CA3 region. (J) Merge of mCherry, NET, and DAPI. (K, L) Colocalization of LC‐originated TH^+^ fibers and NET^+^ fibers. (M, Q) After CNO injection in CA3, green fluorescence indicating c‐Fos in the LC. (N, R) Red fluorescence indicating LC TH^+^ neurons. (O, P, S, T) Colocalization of TH^+^ neurons with c‐Fos. (U) Increased colocalization of TH^+^ neurons with c‐Fos^+^ neurons in the SNI + M3 + CNO group compared with the SNI + M3 + NS group. In C‐E, M‐O and Q‐S, scale bar = 100 μm; In G‐K, scale bar = 50 μm. 4 V: Fourth ventricle. *n* = 6. **p < 0.01.

Adult TH‐cre transgenic mice (20–25 g) were selected and chemogenetics‐rAAV (rAAV‐Ef1α‐DIO‐hM3D(Gq)‐mCherry‐WPREs) was injected into the LC (Figure 4A,B). After 3 weeks of viral transfection, brains were harvested after perfusion for immunofluorescence staining to observe the expression of hM3D(Gq) (Figure 4C–L). Results demonstrated efficient and successful transfection of LC TH neurons with the chemogenetics‐rAAV (Figure 4E,F). Simultaneously, conspicuous red fluorescent neuronal fibers were observed in the mouse hippocampal CA3 region, co‐localizing with NET (Figure 4G–L). Thus, these results confirmed that the LC noradrenergic neurons project to the CA3 region. Following the stable transfection of LC TH neurons in TH‐cre mice with the rAAV, the SNI pain model was established in mice. Subsequently, c‐Fos expression was observed by immunofluorescence of the LC region in both groups after the injection of CNO (0.2 μL, 1 μg/μL) or NS (0.2 μL) into the CA3 region of the hippocampus bilaterally (Figure 4B,M–T). In the SNI + M3 + CNO group, c‐Fos expression increased in the LC TH^+^ neurons (Figure 4U).

### 3.5 Activation of the LC‐CA3 Neurons With Gq‐DREADD in SNI Mice

**FIGURE 5:**
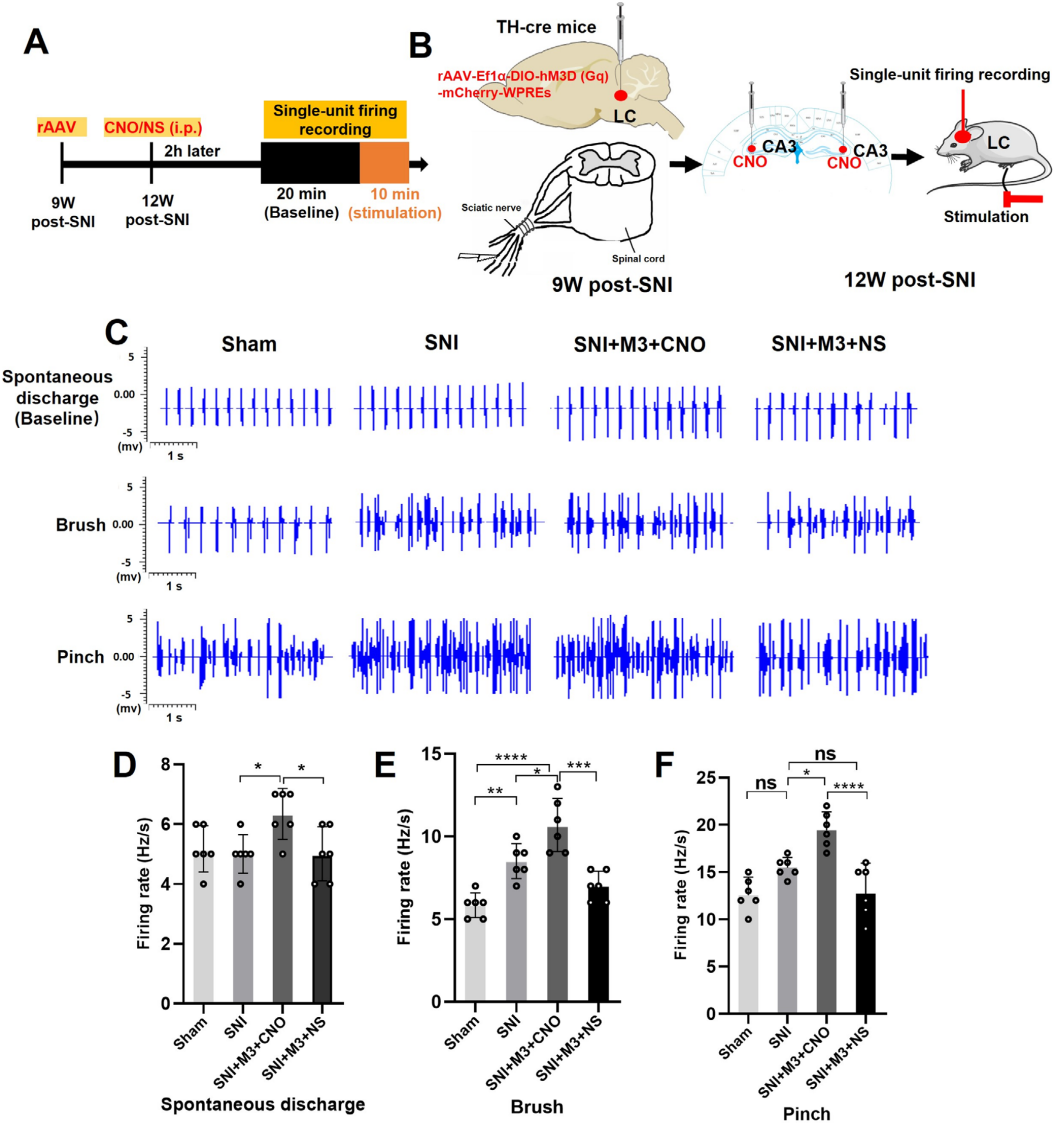
Activating LC‐CA3 neurons increased the discharge frequency of LC neurons in SNI mice. Manipulating the firing rate of LC neurons with Gq‐DREADD. (A, B) Schematic of the Gq‐DREADD strategy and electrophysiological recordings of SNI‐ipsilateral LC neurons 12 weeks post‐SNI. (C) Experimental protocol for non‐noxious brush or noxious pinch applied to the ipsilateral hind paw during CA3 CNO application. (D) In the SNI + M3 + CNO group compared with the other groups, the spontaneous discharge frequency of LC neurons increased. (E, F) Following brush or pinch stimulation, the baseline level was elevated, and compared to the Sham group, the firing frequency of LC neurons in the SNI + M3 + CNO group was significantly increased. Changes in the firing rate of neurons in LC with different stimuli. *p < 0.05, ***p < 0.001, ****p < 0.0001. ns = not significant.

We conducted extracellular single‐unit recordings of LC neurons to assess how spontaneous and noxious stimulation affected discharge frequencies in sham‐operated and SNI mice 12 W post‐SNI. To activate LC, CNO was injected in CA3 (0.5 μL, 1 μg/μL); the SNI + M3 + CNO group showed a higher baseline discharge frequency compared with Sham, SNI, and SNI + M3 + NS groups (Figure 5A–D). Furthermore, following noxious stimulation of the hind paw, LC neuron discharge frequencies significantly increased in the 3 groups compared with baseline discharge frequencies. Additionally, upon stimulation of the SNI‐ipsilateral hind paw with a brush or pinch, the discharge frequency of LC neurons was markedly elevated in both the SNI group and the SNI + M3 + CNO group compared with the Sham group. In contrast, the discharge frequency in the SNI + M3 + CNO group was significantly higher relative to both the SNI and SNI + M3 + NS groups (Figure 5E,F).

### 3.6 Fiber Photometry Recording of NE Release in the Hippocampal CA3 of SNI Mice Under Stimuli, and Hippocampal NE Detection With ELISA After Gq‐DREADD

**FIGURE 6:**
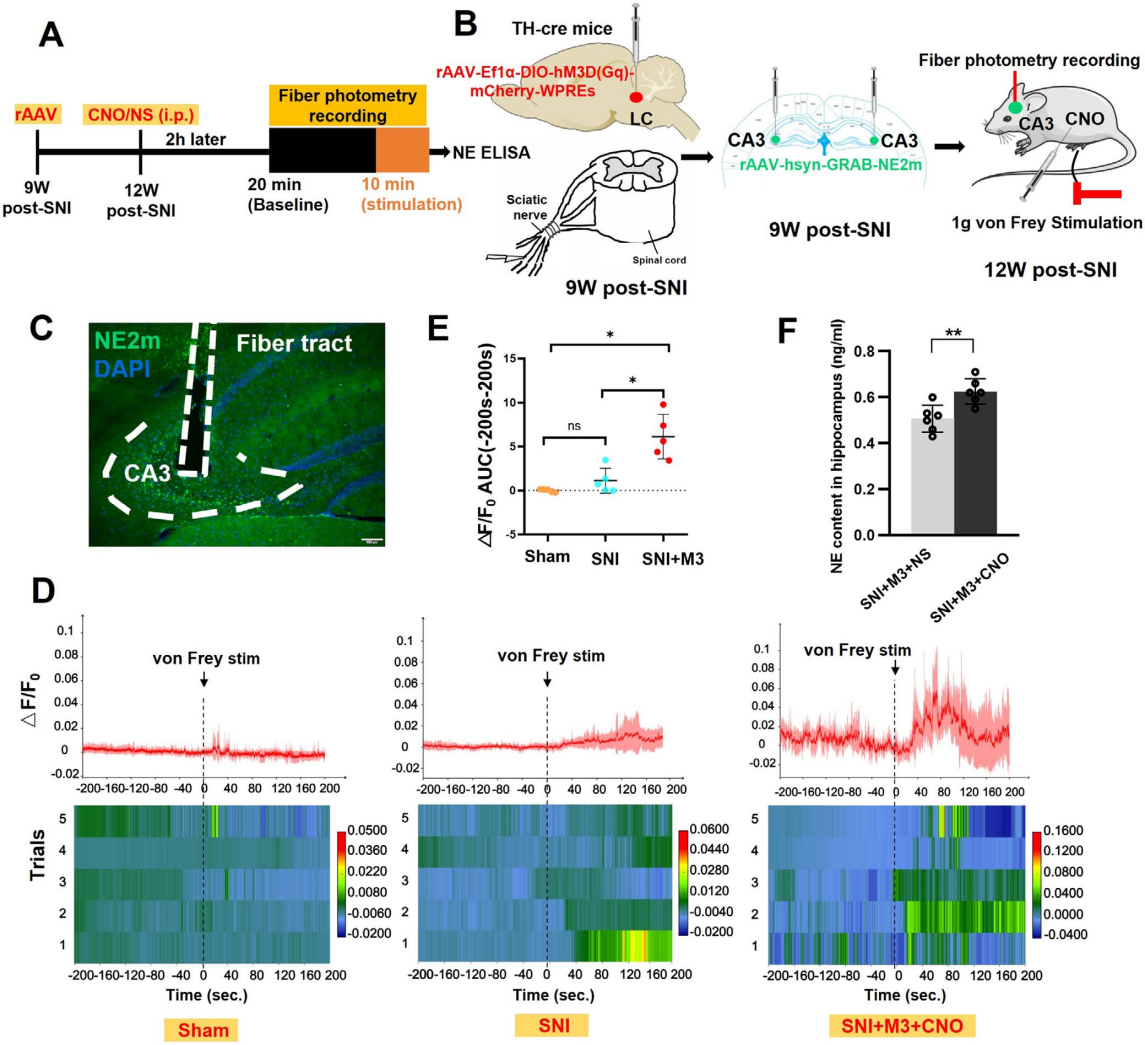
Fiber photometry and NE ELISA revealed that Gq‐DREADD increased NE release in the hippocampal CA3 of mice with chronic NP. (A, B) Experimental protocol for rAAV injection bilaterally at the LC and fiber photometry recording at the SNI‐ipsilateral CA3. (C) The immunofluorescence detecting optical fiber was located in the hippocampal CA3, where the rAAV successfully transfected cells, which was evidenced by NE2m expressing in CA3. (D) The average traces (mean ± 95% CI) of GRAB_NE2m_ signals on hippocampal CA3 neurons under 1 g von Frey stimulation. The fluorescence of GRAB_NE2m_ in CA3 increased in the SNI + M3 + CNO group compared with the SNI + M3 + NS group 12 weeks post‐SNI under 1 g von Frey stimuli. (E) A significantly greater AUC in the SNI + M3 + CNO group compared with the Sham or SNI group. *n* = 5. (F) NE content detected with ELISA in the SNI‐ipsilateral hippocampus showed a significant increase in the SNI + M3 + CNO group compared with the SNI + M3 + NS group 12 weeks post‐SNI under 1 g von Frey stimuli. *n* = 6, *p < 0.05, **p < 0.01. ns = not significant.

Using the genetically encoded G‐protein‐coupled receptor activation‐based NE (GRAB_NE_) sensor [54], we monitored NE signal dynamics in the SNI‐ipsilateral hippocampal CA3 region to confirm the effects of Gq‐DREADD in mice 12 W post‐SNI, during LC activation after CNO injection intraperitoneally, and following 1 g von Frey filament stimulation of the SNI‐ipsilateral plantar of TH‐cre SNI mice (Figure 6A,B). To verify the experimental setup, immunofluorescence was performed to confirm the precise localization of the implanted optical fiber in the hippocampal CA3 (Figure 6C). The results demonstrated that 1 g von Frey stimulation elicited no significant change in GRAB_NE_ fluorescence intensity in both the SNI and Sham groups. However, a marked increase in NE2m fluorescence was observed in the SNI + M3 + CNO group compared with the other two groups (Figure 6D). Quantitative analysis of response magnitude, assessed as the area under the curve (AUC), revealed a significantly greater AUC in the SNI + M3 + CNO group compared with the Sham group (Figure 6E). NE content was detected with ELISA in the SNI‐ipsilateral hippocampus, which showed a significant increase in the SNI + M3 + CNO group compared with the SNI + M3 + NS group 12 weeks post‐SNI (Figure 6F).

### 3.7 Activation of LC‐CA3 Neurons Reduced Anxiety‐ and Depression‐Like Behavior in SNI Mice, Which Could Be Offset by Yohimbine

**FIGURE 7:**
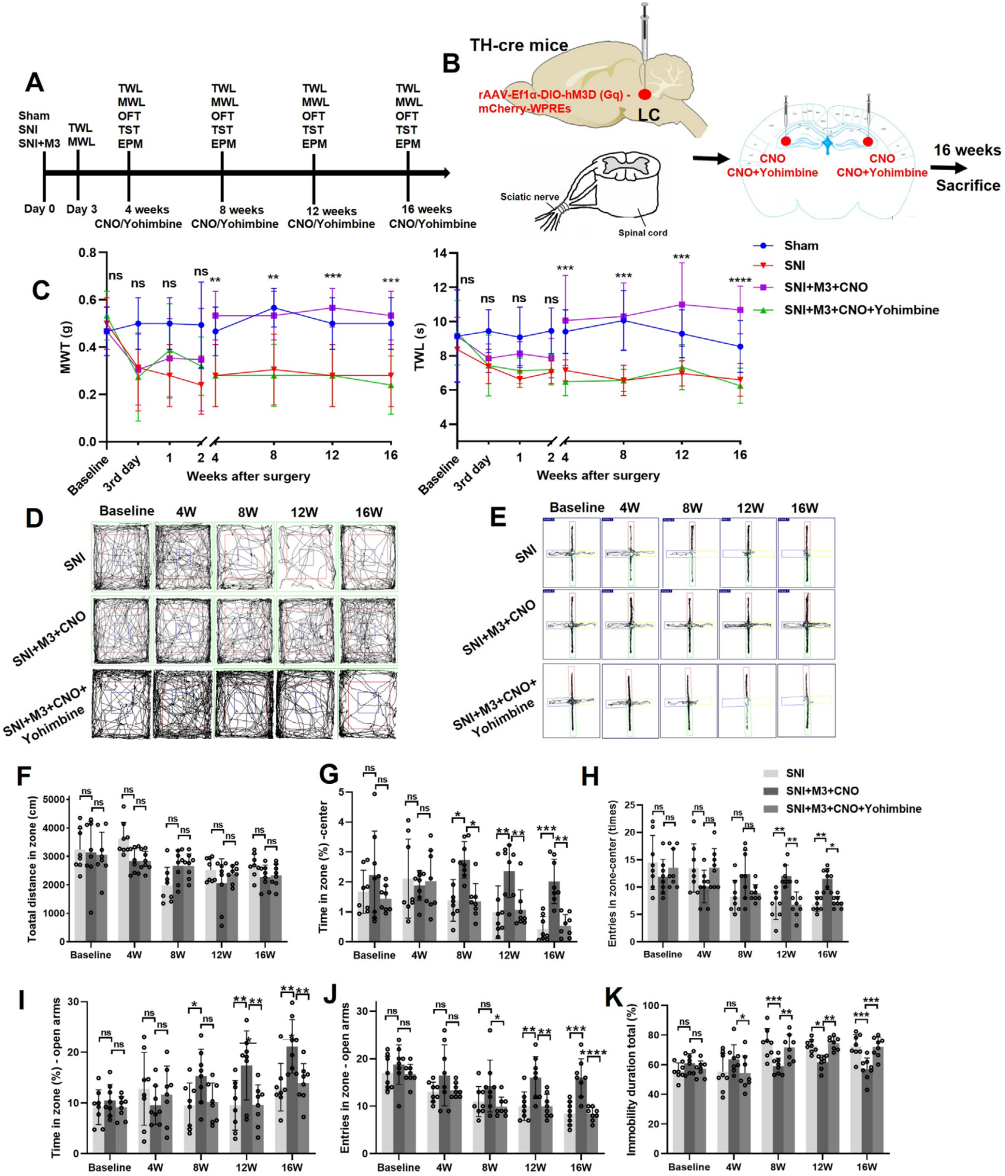
Activation of LC‐CA3 neurons alleviated NP and anxiety‐ and depression‐like behavior in mice with chronic NP, which could be offset by yohimbine. (A, B) Experimental protocol for rAAV injection, establishment of the SNI model and timeline of behavioral observations. (C) Changes of MWT and TWL. Between SNI + M3 + CNO and SNI + M3 + CNO + Yohimbine group, *p < 0.05, **p < 0.01, ***p < 0.001, ****p < 0.0001. ns = not significant. (D, E) Behavior tracks in mice of SNI + M3 + CNO and SNI + M3 + CNO + Yohimbine group in the OFT and the EPM experiments. (F) Total distance covered by mice in the OFT. (G) Percentage of time spent in the center of the OFT and number of entries into the center. (H‐J) EPM time spent in the open arm and number of entries into the open arm. (K) Immobility time during the last 4 min of the TST. *n* = 8. *p < 0.05, **p < 0.01, ***p < 0.001, ****p < 0.0001. ns = not significant.

NE exerts inhibitory effects on microglial overactivation and neuroinflammation through its action on α2‐adrenergic receptors (α2‐ARs) in microglia [22, 55]. Our previous findings demonstrated that yohimbine, a selective α2‐AR antagonist, effectively reverses the analgesic effects induced by LC activation [15]. Building on this discovery, we investigated the behavioral and molecular consequences of intra‐CA3 yohimbine administration in SNI mice with activated LC‐CA3 projections. In the present study, we injected yohimbine into the CA3 of SNI mice with activated LC‐CA3 pathway and observed the behavioral and molecular changes. Yohimbine was injected into the CA3 5 min before CNO administration (3 μg/μL in 20% DMSO in NS) in chronic NP mice (4, 8, 12, and 16 weeks post‐SNI) (Figure 7A,B). The results indicated that the activation of the LC‐CA3 projection in SNI mice could ameliorate NP, as evidenced by significant increases in both MWT and TWL (Figure 7C). In the OFT, there was no significant difference in the total distance between the SNI + M3 + CNO group and the SNI + M3 + CNO + Yohimbine group from 4 to 16 weeks post‐SNI surgery (Figure 7D,F). Starting from 8 weeks post‐SNI surgery, the SNI + M3 + CNO group exhibited an increased percentage of time spent in the center of the open field compared with the SNI + M3 + CNO + Yohimbine group mice (Figure 7D,G), along with an increased number of entries into the center area (Figure 7D,G). The EMT showed that at 12 and 16 weeks post‐SNI surgery, the percentage of time spent in the elevated open arms of the SNI + M3 + CNO group increased compared with the SNI + M3 + CNO + Yohimbine group (Figure 7E,I,J), as well as the number of entries into the center of the elevated maze (Figure 7E,H). These findings suggested that anxiety developed in the SNI + M3 + CNO + Yohimbine group after SNI, while the SNI + M3 + CNO group did not exhibit anxiety. Notably, starting at 8 weeks post‐SNI, the TST results revealed an increased proportion of immobility time in the last 4 min for the SNI + M3 + CNO + Yohimbine group mice compared with the SNI + M3 + CNO group (Figure 7K).

### 3.8 Activation of LC‐CA3 Neurons Attenuated Hippocampal Neuroinflammation in SNI Mice, Which Could Be Offset by Yohimbine

**FIGURE 8:**
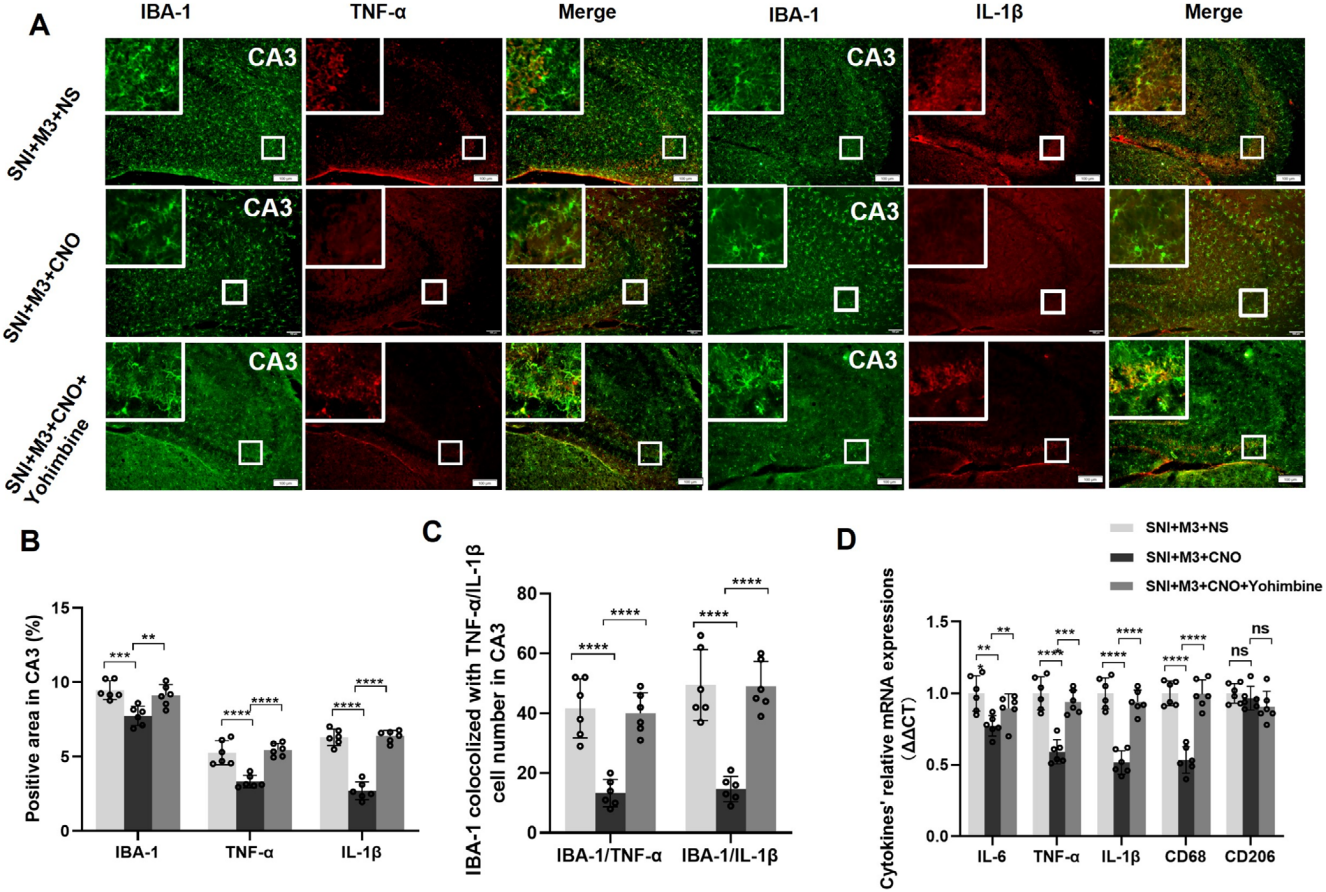
LC‐CA3 activation reduced CA3 neuroinflammation in 12 W post‐SNI mice, which could be offset by yohimbine. (A) Immunofluorescence staining of IBA‐1, TNF‐α, and IL‐1β in the CA3 region of the hippocampus for mice in the SNI + M3 + NS, SNI + M3 + CNO, and SNI + M3 + CNO + Yohimbine groups. (B) Quantification of the relative expression of IBA‐1, TNF‐α, and IL‐1β in CA3. (C) Colocalization of IBA‐1 with TNF‐α or IL‐1β in CA3. (D) Microglia related cytokine mRNA expression in the three groups. Scale bar = 100 μm. *n* = 6, **p < 0.01, ***p < 0.001, ****p < 0.0001. ns = not significant.

At 12 weeks post‐SNI, immunofluorescence was performed to observe neuroinflammation markers such as IBA‐1, TNF‐α, and IL‐1β in CA3 of the SNI + M3 + NS, SNI + M3 + CNO, and SNI + M3 + CNO + Yohimbine groups (Figure 8A). The results revealed a significant reduction of IBA‐1, TNF‐α, and IL‐1β in CA3 of mice of the SNI + M3 + CNO group compared with the SNI + M3 + NS and SNI + M3 + CNO + Yohimbine groups (Figure 8A,B). Additionally, there was a notable reduction in the number of microglia that co‐express TNF‐α or IL‐1β (Figure 8A,C). Subsequent RT‐qPCR analysis assessed mRNA expression levels of inflammatory cytokines in the hippocampus of 12 weeks post‐SNI mice with LC‐CA3 activation. The results indicated that hippocampal mRNA expression levels of inflammatory cytokines, including IL‐6, TNF‐α, IL‐1β, and CD68, were lower in the SNI + M3 + CNO group than those in the SNI + M3 + NS and SNI + M3 + CNO + Yohimbine groups (Figure 8D). In summary, these findings suggest that activation of the LC‐CA3 projection may attenuate abnormal microglial activation in the CA3 region in chronic NP mice, thereby mitigating hippocampal neuroinflammation.

## 4 Discussion

This study established novel temporal associations between chronic NP (16 weeks post‐SNI) and LC neurodegeneration—a pathological progression previously undocumented in NP models. From 8 to 16 weeks post‐SNI, chronic NP induced neuron loss in the LC, suggesting that NE deficiency may be the cause of pain and its comorbidities, that is, anxiety and depression. To test if LC‐CA3 noradrenergic activation effects on NP and anxiety‐ and depression‐like behaviors, we firstly confirmed our hypothesis that prolonged NP induces LC dysfunction and even results in neuron loss. Additionally, a decline in NE content was observed, which was concomitant with the manifestation of depressive symptoms in the SNI mice. Then we confirmed that NP induced activation of microglia and elevated neuroinflammation in CA3. Using Gq‐DREADD, we selectively activated noradrenergic LC‐CA3 projection in 12 weeks post‐SNI mice. Upon specific activation of LC‐CA3, we observed a mitigation of pain hypersensitivity in SNI mice. Notably, anxiety‐ and depression‐like behaviors were ameliorated, accompanied by an elevation of NE content in hippocampal CA3. Furthermore, activation of the LC‐CA3 resulted in a reduction of microglial activation and a decrease in neuroinflammation, evidenced by reduced TNF‐α and IL‐1β levels in the hippocampus. These findings collectively suggested that the targeted activation of the LC‐CA3 projection held therapeutic potential in alleviating chronic NP and in improving the anxiety‐ and depression‐like symptoms by attenuating hippocampal neuroinflammation.

In the present study, we selected the SNI mouse model to replicate a long time (16 weeks) NP in mice. Due to the limited reports on NP mouse models over such a long period of pain duration, model selection is particularly important for this study. SNI models, known for inducing robust behavioral and functional alterations, closely mirror the spectrum of neuropsychiatric disturbances observed in advanced neuropathic states, encompassing anxiety, depressive behaviors, and cognitive deficits [56]. Given the multifaceted etiologies contributing to NP, it was imperative to employ an animal model that authentically recapitulates the diverse pathophysiological changes inherent in human patients. Conventional animal models for NP encompass techniques such as peripheral nerve ligation and dissociated innervation [57, 58, 59]. Notably, the SNI model, applied in this experiment, demonstrated sustained changes in mechanical sensitivity and thermal reactivity in rodents for an extended period, up to 12 months [60]. In the present study, SNI mice developed mechanical hypersensitivity within 3 days post‐surgery, peaking at 1 week and maintaining intensity through 16 weeks. Importantly, this heightened pain sensitivity persisted consistently throughout the 16‐week postoperative period, showcasing the SNI model's efficacy in maintaining a prolonged and stable manifestation of NP. This enduring maintenance of NP indicated that the SNI model was particularly well‐suited for investigating the nuanced dynamics of NP onset, progression, and regression. Moreover, the SNI model, employed in this study, yielded not only prolonged NP but also induced neuropsychiatric disorders in mice, including anxiety‐ and depression‐like behaviors [61, 62]. Behavioral assessments conducted at 4‐week intervals postoperatively uncovered the emergence of anxiety‐like behaviors at 8 weeks and depressive behaviors at 12 weeks after SNI. The model's dual fidelity to sensory and behavioral pathology underscored its utility for dissecting NP progression and testing interventions targeting both nociceptive and emotional circuits.

Clinical NP syndromes such as postherpetic neuralgia often exhibit chronic progression over years—a trajectory recapitulated in our SNI model. While acute NP activated LC neurons, the consequences of sustained nociceptive signaling on this vulnerable noradrenergic nucleus [63, 64] remain poorly understood. We identified a significant decrease in LC neurons in SNI mice after 8 weeks post‐SNI, a novel finding that may explain the decrease in NE in the brain and spinal cord due to chronic pain [65].

Immunofluorescence analysis of IBA‐1 expression in the mouse hippocampus at 4 weeks post‐SNI revealed significant microglial activation with proinflammatory polarization. As resident immune sentinels of the CNS, microglia play a pivotal role in orchestrating neuroinflammatory responses through their dynamic functional states. The dichotomous nature of microglial activation, proposed by Chen et al. [66] posited its dual role as both detrimental and protective in neuropathic contexts. Activated microglia exhibited a spectrum of functions, releasing anti‐inflammatory cytokines like IL‐10 [67], while concurrently secreting pro‐inflammatory mediators, notably TNF‐α [15]. Chronic NP induced persistent microglial activation in central pain‐processing regions, including the hippocampus, creating a self‐amplifying cycle of neuroinflammation [68]. Neuroinflammation, serving dual roles of eliminating injury causation and facilitating tissue repair, assumed heightened significance in NP scenarios. However, this defense mechanism was susceptible to devolving into a self‐perpetuating cycle of persistent immune cell activation, contributing to the chronicity of NP [69].

Our findings revealed distinct regional heterogeneity in hippocampal neuroinflammation, with the CA3 subfield demonstrating marked microglial proliferation as evidenced by significantly elevated IBA‐1 fluorescence intensity compared to CA1 and CA2 regions. Immunofluorescence results additionally underscored an augmented area of TNF‐α and IL‐1β‐positive fluorescence across all hippocampal regions in 12‐week post‐SNI mice. Notably, the CA3 area exhibited a significant increase in neuroinflammation compared with CA1 and CA2 areas. One study had implicated the CA3 region in the pathophysiology of NP‐induced depression in 21 days post‐SNL mice [43]. Pathological changes in the present SNI mice corroborated these findings, manifesting evident proinflammatory activation of microglia in the CA3 region of the hippocampus. Collectively, these results underscored the significant involvement of neuroinflammation within the CA3 region in the progression of NP‐associated anxiety and depression.

Prior studies demonstrated LC activation alleviates NP in chronic constriction injury (CCI) models [24]. Building on this, we employed rAAV transfection in the LC of TH‐cre mice, which showed direct projection to the hippocampal CA3 region 3 weeks after AAV injection. Colocalization evaluation with NET in CA3 further confirmed the LC‐CA3 noradrenergic projection. Evidence from prior studies underscored that the CA3 region received a substantial number of projections from the LC, exerting influence not only on the spatial learning abilities and electrophysiological activity of the hippocampus in mice [70], but also on pain perception and mental states [43]. By detecting the colocalization of mCherry that was expressed in LC neurons and NET, a noradrenergic neuron specifically expressing transporter in the CA3, we confirmed that the LC directly and enormously projected to CA3 in 12 weeks post‐SNI mice, indicating that CA3 is one of the main destinations of NE transmission.

Our findings demonstrated that chemogenetic activation of LC‐CA3 noradrenergic projections remarkably ameliorated pain hypersensitivity in chronic NP (12 weeks post‐SNI) mice, concurrently preventing the onset of anxiety‐ and depression‐like behaviors. Existing literature had underscored the intimate connection between anxiety, depression, and the proinflammatory activation of microglia‐induced neuroinflammation, wherein persistent microglial activation often accompanies depressive states [71]. A pertinent study by Zhang et al. [72] shed light on the role of the IL‐4‐driven phenotypic shift in hippocampal microglia, which fostered the growth of neurotrophic factors reliant on brain‐derived neurotrophic factor, thereby contributing to the amelioration of depressive symptoms. In a parallel context, Li et al. [73] delved into the sustained activation of microglia in a spinal cord injury model, employing a colony‐stimulating factor 1 receptor antagonist to deplete microglia in the brain. The subsequent reduction in microglial activation, coupled with the restoration of neuroinflammation in the brain and spinal cord, coincided with improvements in cognition, depressive‐like behavior, and motor function in mice.

The alpha‐2 adrenergic receptors (α2‐ARs) on microglia [32] served as critical regulators of neuroinflammatory responses [15]. NE binding to α2‐ARs activated signaling pathways coupled with inhibitory G proteins (Gi), thereby suppressing the release of pro‐inflammatory cytokines and alleviating neuroinflammation in microglia [22, 74]. Studies have shown that NE, through the action of α2 receptors, decreases the secretion of inflammatory factors and chemokines by microglia, subsequently reducing neuronal inflammatory responses [22, 55]. This mechanism holds significant importance in the treatment of NP and neurodegenerative diseases. Our experimental blockade of CA3 α2‐ARs via yohimbine exacerbated mechanical hypersensitivity and precipitated anxiety/depression‐like behaviors in SNI mice, concurrent with CA3 neuroinflammatory escalation. We speculated that yohimbine injected in CA3 inhibited the α2‐AR in microglia, which offset the anti‐inflammatory effect of NE on microglia. Aligning with these insights, our study demonstrated that the activation of the LC‐CA3 projection pathway resulted in elevated NE content and a concomitant reduction in aberrant microglial activation. This effective alleviation of NP in mice was coupled with a decreased likelihood of anxiety‐ and depression‐like behaviors.

Our immunofluorescence data demonstrated marked TNF‐α and IL‐1β elevation in the hippocampal CA3 of SNI mice. Chemogenetic activation of LC‐CA3 noradrenergic projections significantly attenuated these inflammatory cytokines across hippocampal subregions. Literature had illuminated that microglia express α1, α2, β1, and β2 adrenergic receptors, thus enabling them to be regulated by NE [32]. NE exerted its effects through adrenergic receptor activation and induced an elevation in cyclic adenosine monophosphate levels within microglia, governing their response to injury and inhibiting the production of inflammatory mediators [75].

This study had several limitations that warrant consideration: (1) The other NE receptors, such as β2‐AR [76], which could be effective for NE‐microglia reaction, were not evaluated in the present study. (2) Changes in other brain regions were not observed, such as the PFC [77], which is another brain region that has an effect on both pain and anxiety, although others have reported that activation of the LC‐PFC can cause aversion‐like pain in rats [77]. (3) LC‐CA3 noradrenergic activation might induce dopamine release in CA3, which was not tested in the present study. Investigations had been delved into the intricate network of dopaminergic projections from the LC to the dorsal hippocampus, known to exert a profound influence on spatial learning and memory capabilities [78]. Additionally, LC projections to the CA1 hippocampal region had been implicated in the release of dopamine, modulating chronic stress‐induced depression [79]. This emphasized the predominantly dopaminergic nature of projections from the LC to the hippocampus. (4) Due to the prolonged modeling timeline and potential confounds from hormonal cyclicity in females, only male mice were included. As sex differences critically shaped pain neurobiology [46], our findings are contextually limited to males. Furthermore, given that spontaneous pain represents a key characteristic of NP, incorporating spontaneous pain behavior experiments in future studies would provide a more comprehensive evaluation of the LC‐CA3 noradrenergic pathway's role in NP.

## 5 Conclusion

Our study delineated a neurodegeneration‐neuroinflammation axis in chronic NP. We confirmed that prolonged NP (8–16 weeks post‐SNI) induces LC neuron loss and demonstrated the pivotal involvement of LC‐CA3 noradrenergic projections and microglial proinflammatory activation were underscored by our findings. Microglia within the CA3 region in SNI mice exhibited heightened activation and escalated expression of inflammatory cytokines. Activation of LC‐CA3 noradrenergic neuron with Gq‐DREadD increases hippocampal NE release, which reduces abnormal activation of microglia and neuroinflammation in the hippocampus. These coordinated effects alleviate mechanical hypersensitivity and rescue anxiety/depression‐like behaviors, positioning LC‐CA3 neuromodulation as a dual‐target strategy against NP and its psychiatric sequelae.

## Author Contributions

S.C., H.X., Y.W., and T.Y. conceptualized and designed the study. J.L., S.W., L.M., W.P., C.L., J.Z., and H.Z. conducted experiments and contributed to data acquisition. H.Z., J.L., L.M., X.L., T.Y., J.M., H.X., and S.C. performed data analysis and interpretation. The manuscript was drafted by H.Z. and S.C., with critical revisions from all authors. Visualizations were created by H.Z., S.W., and J.Z. All authors reviewed the final manuscript, provided intellectual input, and approved the submitted version.

## Ethics Statement

All experiments were approved by the Laboratory Animal Welfare & Ethics Committee of Zunyi Medical University (ZMU21‐2210‐002). Animal studies were performed in accordance with the Guide for the Care and Use of Laboratory Animals.

## Consent

The authors have nothing to report.

## Conflicts of Interest

The authors declare no conflicts of interest.

## Data Availability

The data that support the findings of this study are available from the corresponding author upon reasonable request.
